# Multicomponent Crystal of Metformin and Barbital: Design, Crystal Structure Analysis and Characterization

**DOI:** 10.3390/molecules26144377

**Published:** 2021-07-20

**Authors:** Linhong Cai, Lan Jiang, Cong Li, Xiaoshu Guan, Li Zhang, Xiangnan Hu

**Affiliations:** 1 Department of Medicinal Chemistry, School of Pharmacy, Chongqing Medical University, Chongqing 400016, China; cailinhong123@163.com (L.C.); licong11105@126.com (C.L.); 17082343045@163.com (X.G.); zhangli3357@163.com (L.Z.); 2 College of Environment and Resources, Chongqing Technology and Business University, Chongqing 400067, China; jianglan@ctbu.edu.cn

**Keywords:** multicomponent crystal, crystal design, supramolecular synthon, metformin, barbital

## Abstract

The formation of most multicomponent crystals relies on the interaction of hydrogen bonds between the components, so rational crystal design based on the expected hydrogen-bonded supramolecular synthons was employed to establish supramolecular compounds with desirable properties. This theory was put into practice for metformin to participate in more therapeutic fields to search for a fast and simple approach for the screening of candidate crystal co-formers. The prediction of intermolecular synthons facilitated the successful synthesis of a new multicomponent crystal of metformin (Met) and barbital (Bar) through an anion exchange reaction and cooling crystallization method. The single crystal X-ray diffraction analysis demonstrated the hydrogen bond-based ureide/ureide and guanidine/ureide synthons were responsible for the self-assembly of the primary structural motif and extended into infinite supramolecular heterocatemeric structures.

## 1. Introduction

**Figure 1:**
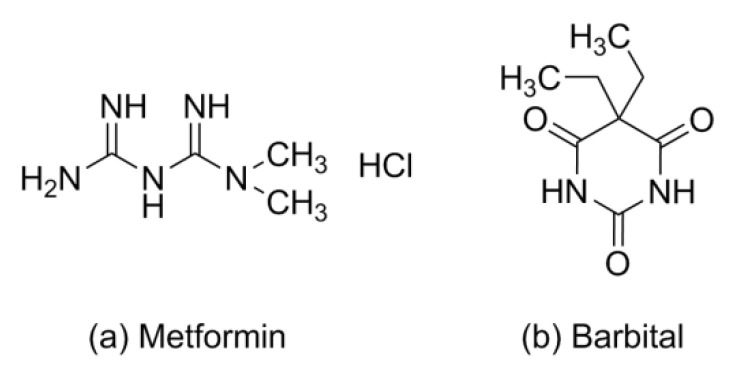
Chemical structures of (**a**) metformin and (**b**) barbital.

Metformin (Met), a first-line oral hypoglycemic drug, is widely used in type 2 diabetes [1]; the chemical structure is shown in Figure 1a. Patients with diabetes usually have a high co-prevalence rate of depression and neurodegenerative diseases such as Parkinson’s disease and Alzheimer’s disease [2,3,4]. The pharmacokinetics show that Met can rapidly arrive at the peak concentration in the cerebrospinal fluid and hippocampus crossing blood–brain barriers within 30 min; it has received a lot of attention in terms of decreasing the oxidative stress of the brain and neuronal apoptosis in patients with neurodegenerative, affective, and metabolic diseases [5,6].

In recent years, more and more attention has been given to the field of multicomponent crystals including salts or cocrystals [7,8] because they are superior to compound preparations and provide an alternative for pharmaceutical combination application [9,10,11]. Through the action of ionic bonds or non-covalent bonds, active pharmaceutical ingredients (APIs) and crystal co-formers (CCFs) are combined into one unit cell without changing the covalent structure of the drug molecule, which may not only improve druggability, stability, and bioavailability by altering the solubility, moisture absorption, and other physicochemical or mechanical properties of APIs, but also interact with different drug targets, causing a synergistic effect between drugs, a new choice for solid pharmaceutical preparations [12,13,14,15].

Multicomponent crystals represent the application of supramolecular concepts in the pharmaceutical industry [16]. In supramolecular chemistry, a supramolecular compound is defined as a product of intermolecular recognition and self-assembly through hydrogen bonds or van der Waals forces, π–π stacking, electrostatic attraction, and other non-covalent bonds connecting active pharmaceutical ingredients with other organic molecules [17,18]. Among these interactions, hydrogen bonds are the most important force in the formation of supramolecular compounds due to their high strength, directionality, saturation, and multiple bonding modes [19]. Accordingly, the rational design of multiple component crystals is usually based on hydrogen-bonded supramolecular synthons [20].

**Figure 2:**
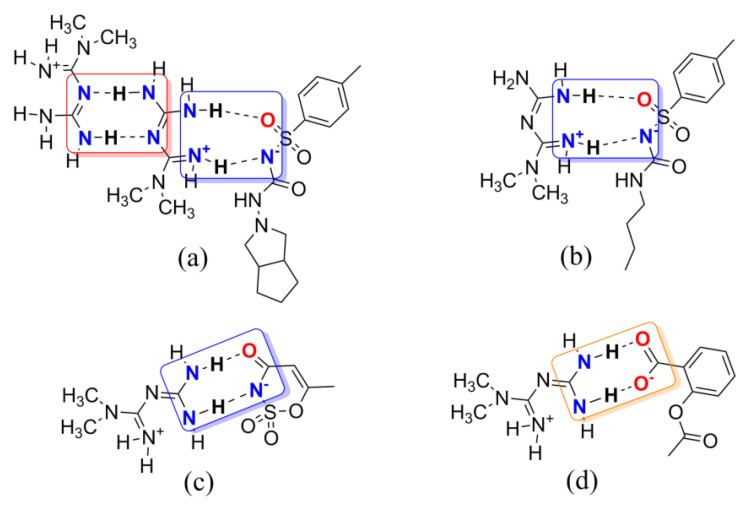
Several examples of multicomponent chemical structures: (**a**) gliclazide metformin; (**b**) amino-methaniminium(butylcarbamoyl)(4-methylbenzene-1-sulfonyl)azanide; (**c**) metformin acesulfame; (**d**) amino[(diaminomethylidene)amino]-*N*,*N*-dimethylmethaniminium 2-(acetyloxy)benzoate. Supramolecular homo- and/or heterosynthons A, B, and C are highlighted with red, blue, and orange boxes, respectively.

Based on the synthon theory, Met can introduce a CCF to be a new entry with intellectual property rights used in the central nervous system. Therefore, a set of multicomponent crystal structures of metformin was extracted from the Cambridge Structural Database (CSD). As drawn in Figure 2, two Met molecules form a centrosymmetric dimer structure through the N–H···N hydrogen bonds based on homosynthon A [15]. The imine groups of Met can also interact with sulfonyl groups of gliclazide or tolbutamide or amide groups of acesulfame via the N–H···O and N–H···N hydrogen bonds building heterosynthon B to establish intermolecular interactions [12,21]. In addition, metformin can also construct supramolecular heterodimers with deprotonated aspirin via synthon C [13]. Not only that, the introduction of CCFs has changed the way of connection and stacking between metformin molecules, showing better physicochemical and pharmaceutical properties, and significantly improved moisture absorption and stability [12,13,15,21]. In the existing literature, metformin has been proven to be a promising co-former with multiple hydrogen bond donors and acceptors during the formation of multicomponent crystals, making it suitable as an API for this experiment.

**Figure 3:**
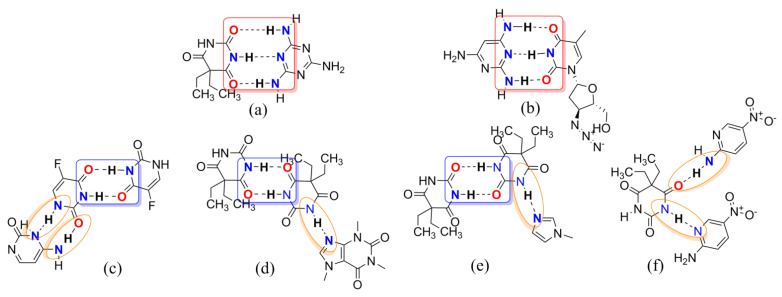
Several examples of multicomponent chemical structures: (**a**) melamine barbital; (**b**) bis(zidovudine) 2,4,6-triaminopyrimidine; (**c**) 4-amino-2-oxopyrimidine 2,4-dioxo-5-fluoropyrimidine; (**d**) bis(barbital)–caffeine complex; (**e**) bis(barbital) 1-methylimidazole; (**f**) 5,5-diethylpyrimidine-2,4,6(1*H*,3*H*,5*H*)-trione bis(5-nitropyridin-2-amine). Supramolecular homo-and/or heterosynthons I, II, and III are highlighted with red, blue, and orange boxes, respectively.

Barbiturates, derivatives of the barbituric acid, were commonly used as sedative–hypnotics in the mid-twentieth century [22]. In 1903, diethyl barbituric acid (barbital) was created as the first barbiturate with central nervous system inhibitory effects [23]. Barbital (Bar) is mostly infrequently used in human medical treatment today but is still listed in pharmacopeias (e.g., in the European and Japanese Pharmacopeias) [24]. Barbital is characterized by symmetry, small molecular weight, and multiple hydrogen bonding sites, which makes it easy as a model CCF to generate some supramolecular compounds, as shown in Figure 1b. In Figure 3, barbital and melamine, which features an equally high symmetry, form a strong hydrogen bond through the three-point synthon I [25]. The supramolecular compound formed between 2,4,6-triamino-pyrimidine and zidovudine, which also has the groups of acylurea, further confirmed the rational existence of synthon I [26]. Barbital can also form homodimers like metformin through two-point supramolecular synthon II, and then another nitrogen–hydrogen bond interacts with another molecule containing an N atom to form N–H···N and/or N–H···O interactions based on synthon III, similar to the hydrogen bonding of 5-fluorouracil [27,28,29,30].

We, therefore, postulated that guanidine/ureide-based supramolecular assembly would employ supramolecular homo- or heterosynthons for constructing hydrogen bonds with other supramolecular reagents. Consequently, by allowing APIs containing guanidino groups to associate with a co-former involving the –(CONHCO)– group, we surmised that guanidino and acylurea moieties would organize supramolecular synthon assemblies formed via N–H···O and N–H···N hydrogen bonds into extended architectures. It was expected, therefore, that Met and/or Bar were able to form a homodimer through synthon A and I, respectively, and then linked with each other via synthon B or synthon III to form a 1D chain.

To test our design strategy, the anion exchange reaction was employed, and the resulting crystal was determined primarily by single crystal X-ray diffraction (SCXRD). The other characterization methods included powder X-ray diffraction (PXRD), Fourier-transform infrared spectroscopy (FT-IR), thermogravimetric analysis (TGA), differential scanning calorimetry (DSC), and ^1^H NMR spectroscopy analysis. Dynamic vapor sorption (DVS) was conducted to measure the moisture sorption behavior of multicomponent crystals.

## 2. Results and Discussion

### 2.1. Single Crystal X-ray Diffraction Analysis of the Crystal Structure

**Figure 4:**
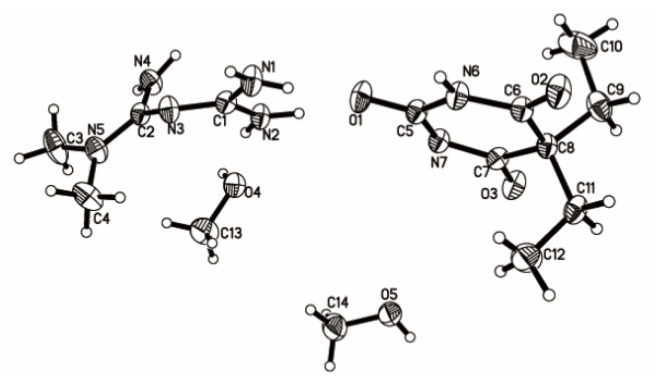
Thermal ellipsoid figure drawn at the 50% probability level.

Single crystalline materials of 1:1 Met–Bar isolated by cooling crystallization of 1,1-dimethyl-biguanidhydrochloride and 5,5-diethylbarbituric acid sodium (1:1) from methanol were suitable for analysis by SCXRD. The corresponding crystallographic data and refinement details are summarized in Table 1. It crystallized in the triclinic space group P-1: a = 7.3409(7) Å, b = 11.1698(10) Å, c = 13.0980(9) Å, α = 74.680(7)°, β = 78.624(7)°, γ = 84.157(8)°, Z = 2, and the final R1 was 0.0525 (I > 2σ(I)) and wR2 was 0.1552 (all data). There were one molecule of Met, one molecule of Bar, and two molecules of methanol in an asymmetric unit in Figure 4. The N–H hydrogen atom of the malonylurea group of Bar transferred to the imine group of Met, which proved that this was a salt-type multicomponent crystal.

**Figure 5:**
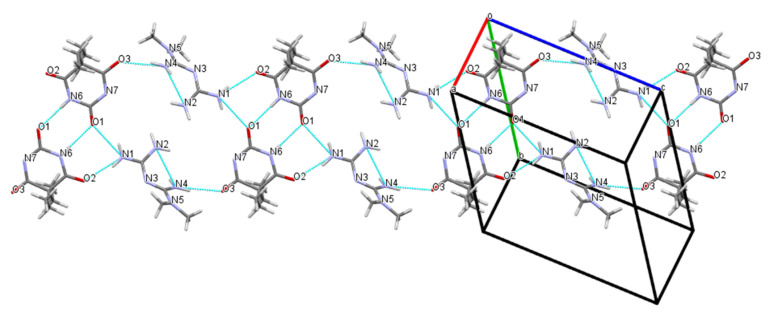
One-dimensional layered structure connected by hydrogen bonds of Met–Bar.

As shown in Table 2 and Figure 5, the crystal structure generated a centrosymmetric dimeric unit of the Bar molecule assembled by the expected amide–amide homosynthon II with the N6···O1 distance of 2.931 Å. The Met molecule linked two Bar molecules into an infinite one-dimensional (1D) chain via N(4)–H(4D)···O(3) and N(1)–H(1A)···O(2) hydrogen bonding interactions with the N4···O3 and N1···O2 distances of 3.057 and 2.967 Å, respectively. The N–H···O hydrogen bonds created a 1D rod motif by the predicted one-point synthon III instead of forming a centrosymmetric 0D dimer through synthon A between the Met molecules. Adjacent antiparallel chains were arranged into ribbons through pairs of symmetry between the related Bar homodimeric motif and the N(1)-H(1B)···O(1) hydrogen bond; r(N1···O1), 2.939 Å; ∠(N–H–O), 142°. In addition, the two eight-membered rings formed through the connectivity of the N(1) atom of Met and the O(1) atom of Bar further stabilized the crystal structure together with a homodimer ring of Bar. Based on the intermolecular interactions described above, all donor and acceptor moieties in Bar except for the ionized N(7) atom generated hydrogen bonds with Met molecules or Bar itself. This indicated that Bar could be a promising crystal co-former during the formation of multicomponent crystals.

**Figure 6:**
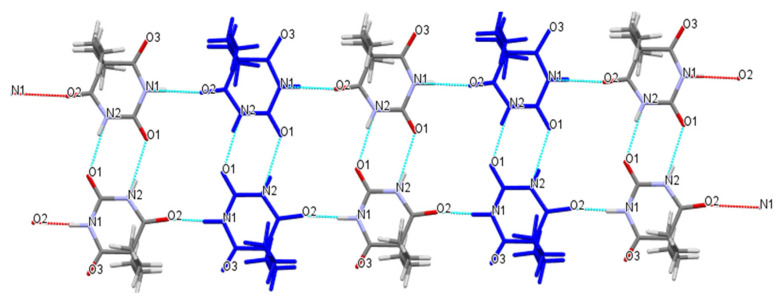
Cooperative N–H···O hydrogen-bonded linear chain supramolecular homosynthons exhibited in the crystal structure of barbital.

Compared with the pure crystal structure of Bar extracted from the CSD in Figure 6 [31], the multicomponent crystal structure reassembled the packing arrangements of the structure at the molecular level in that the blue 5,5-diethylbarbituric acid molecules were substituted with metformin molecules (Figure 4). Met–Bar was thereby sustained through supramolecular hydrogen-bonded homo- and heterosynthons (synthons II and III) in such a manner that a heterocatemeric chain similar to the homomeric catemer present in pure 5,5-diethylbarbituric acid was generated. To summarize, the rational design of this multicomponent crystal based on supramolecular synthon analysis worked very well, and the expected synthons II and III were observed in the crystal structure sustained by a supramolecular heterocatemer.

### 2.2. Characterization of the Crystal Structure

**Figure 7:**
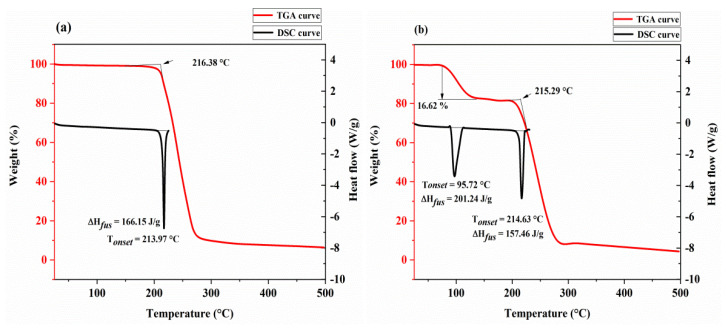
Thermal analysis of the desolvate (**a**) and the solvate (**b**) of Met–Bar. The red and black lines represent the TGA and DSC curves, respectively.

The thermal behavior was analyzed using the TGA and DSC techniques. We obtained the weight change values of the substance converted to the percentage by weight within a specific heating temperature range of 25–500 °C, as drawn in Figure 7 (represented by the red lines). In the unit cell diagram of the monocrystalline solid, as shown in Figure 4, we observed that the multicomponent crystal contained two methanol molecules, which was consistent with the TGA profile of the solvate with a continuous weight loss of 16.62% before melting (theoretical weight loss, 16.7%) in Figure 7b. However, after the solvate was dried under vacuum at 55 °C, TGA demonstrated that when Met–Bar disintegrated at 216.38 °C, there was no significant downward tendency of weight (%) from 25 °C to 216.38 °C. It proved that our multicomponent crystal had undergone a crystal transformation phenomenon. The prepared Met–Bar had been transformed from a solvate to a desolvate and further identified by DSC and ^1^H NMR spectroscopy analysis. Unfortunately, the single crystal of the desolvate could not be obtained.

As drawn in Figure 7a, consistent with the TGA thermogram of the desolvate, there was only a relatively prominent endothermic peak; no endothermic peak caused by endothermic desolvation which was different from the endothermic peak of the solvate at 95.72 °C due to desolvation in Figure 7b was observed. In the DSC curve of the desolvate, the intersection of the maximum slope of the front of the peak and the baseline extension line was the melting point of Met–Bar (213.97 °C), which was much higher than that of Met (112.5 °C) [12,13] and Bar (190 °C) [32] but lower than that of Met·HCl (223–226 °C). Combined with the TGA thermogram, it was proved that the melting of Met–Bar occurred before decomposition at 216.38 °C.

**Figure 8:**
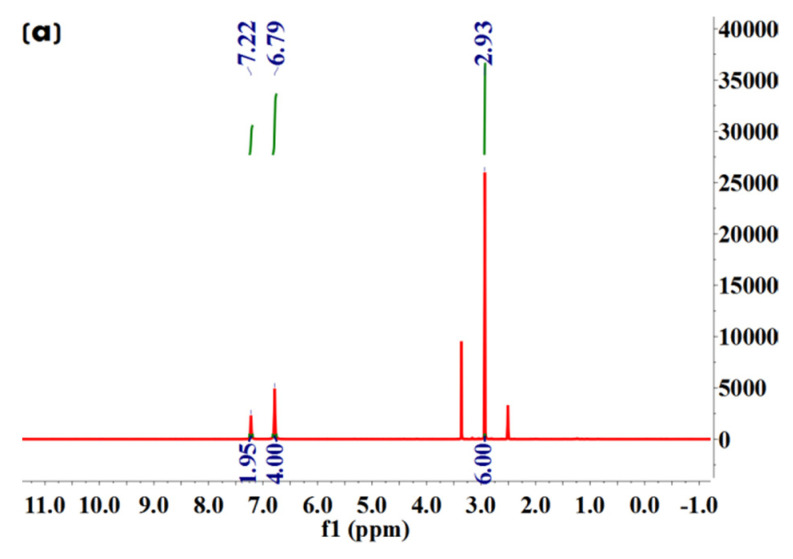

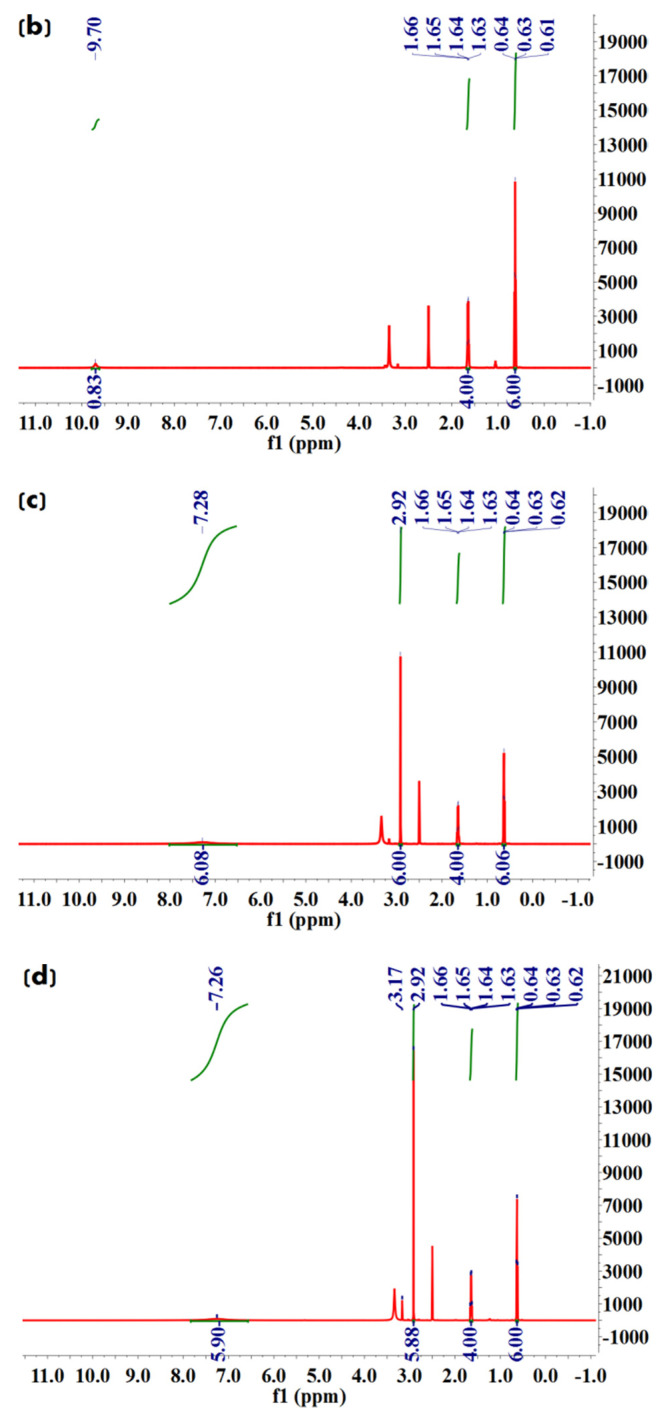
^1^H NMR spectra of Met·HCl (**a**), Bar·Na (**b**), multicomponent crystal of Met–Bar (**c**), and the solvate crystal (**d**).

We further conducted ^1^H NMR spectroscopy analysis to characterize molecular structures of Met·HCl, Bar·Na, and the solvate and desolvate samples of Met–Bar. In Figure 8c, since the two methyl groups in Met and the two ethyl groups in Bar did not participate in the formation of hydrogen bonds, their chemical shifts did not change in the salt-type crystalline compound, which were 2.92, 1.64, and 0.63, respectively. Due to the occurrence of the ion exchange reaction, sodium chloride was removed to form a new ionic bond, and the N–H bond in Met participated in the formation of hydrogen bonds, increasing chemical shifts in the multicomponent crystal, which appeared at 7.28, a single absorption peak. However, owing to the influence of the water peak of DMSO-*d*_6_, the active hydrogen atom with a chemical shift of 9.70 on Bar disappeared. Additionally, the methanol peaks were shown at 3.16 in the solvate crystal in Figure 8d but did not appear in the desolvate samples. It was proved that the crystal was a methanol-free compound whose stoichiometric ratio of Met and Bar was 1:1, as shown in Figure 8. Compared to the commonly applied doses of Met and Bar, this constant ratio of this form of APIs might be possible to administer as a drug. The general oral dosage of Met, marketed under the trade name Glucophage, was 0.5 g twice daily or 0.85 g once daily with meals. The daily dosage was increased by 0.5 g at weekly intervals or by 0.85 g at biweekly (every 2 weeks) intervals up to a maximum of 2.55 g daily given in divided doses [33]. Barbital was marketed by Bayer as Veronal, and 3.5–4.4 g was the deadly dose, but sleep had also been prolonged up to ten days with recovery [34]. The doses used range from 0.25 g to 3.5 g to produce weak, moderate, or strong effects; the therapeutic dose frequently was 0.6–1.0 g; a dose of 0.5 g sufficed to produce sleep within an hour [35]. From this point of view, the possible use of 0.5 g Met–Bar once or twice a day could be administered based on this 1:1 constant ratio.

The ^1^H NMR data of Met·HCl, Bar·Na, multicomponent crystal of Met–Bar, and the solvate crystal, respectively, were:

^1^H NMR (600 MHz, DMSO-*d*_6_) δ 7.22 (s, 2H), 6.79 (s, 4H), 2.93 (s, 6H).

^1^H NMR (600 MHz, DMSO-*d*_6_) δ 9.70 (s, 1H), 1.64 (q, *J* = 7.4 Hz, 4H), 0.63 (t, *J* = 7.4 Hz, 6H).

^1^H NMR (600 MHz, DMSO-*d*_6_) δ 7.28 (s, 6H), 2.92 (s, 6H), 1.64 (q, *J* = 7.4 Hz, 4H), 0.63 (t, *J* = 7.4 Hz, 6H).

^1^H NMR (600 MHz, DMSO-*d*_6_) δ 7.26 (s, 6H), 2.92 (s, 6H), 1.64 (q, *J* = 7.4 Hz, 4H), 0.63 (t, *J* = 7.4 Hz, 6H).

**Figure 9:**
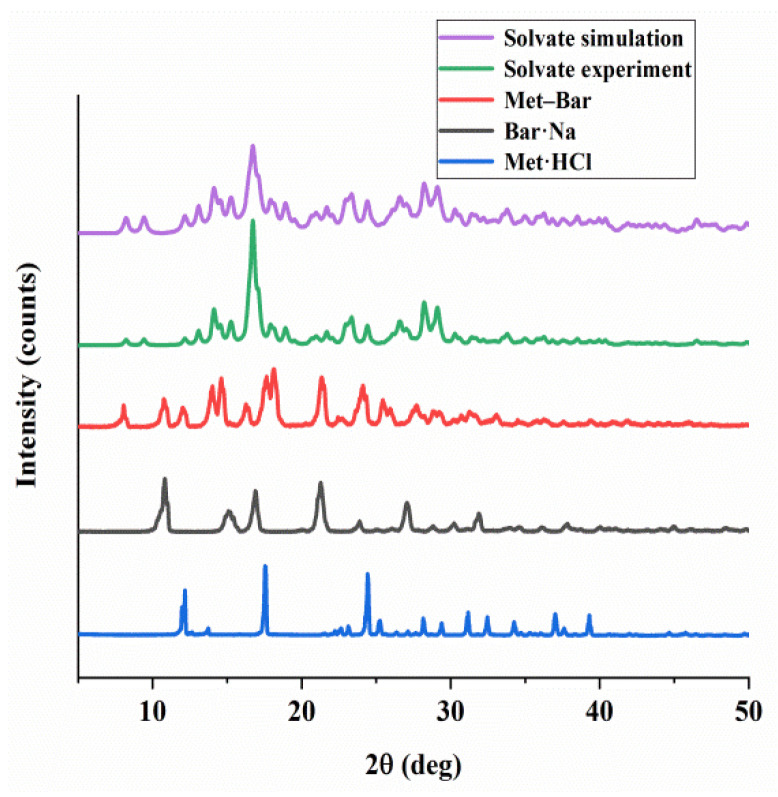
Powder X-ray diffractograms of Met·HCl, Bar·Na, and Met–Bar.

Powder X-ray diffraction had much broader applications than single crystal X-ray diffraction, which could quickly identify the structural difference between our raw materials and Met–Bar, and the results are shown in Figure 9. The diffraction peaks (14.08°, 16.8°, 23.36°, 24.48°, 28.3°, 29.1°) of the experimental PXRD pattern of the solvate matched the simulated PXRD pattern based on SCXRD well, showing that the prepared crystals were of pure crystalline phases. Additionally, the characteristic diffraction peaks of Met·HCl with 2θ were at 12.6°, 17.54°, 24.42°, 31.16°, 32.46°, and Bar·Na was characterized with 2θ at 10.82°, 15.08°, 16.88°, 21.28°, 27.06°, 31.9°. Obviously, the diffraction peaks of Met–Bar (8.04°, 10.76°, 12.0°, 14.6°, 18.12°, 21.34°, 24.1°) were different from those of the two raw materials and the solvate crystal because there were completely different and new peaks emerging at 8.04°, 14.02°, 14.6°, etc., indicating that the PXRD pattern of Met–Bar was not a simple superposition of the patterns of Met·HCl and Bar·Na. Met–Bar was an absolutely new compound, not a mixture of the two raw materials, which was consistent with the FT-IR spectrum.

**Figure 10:**
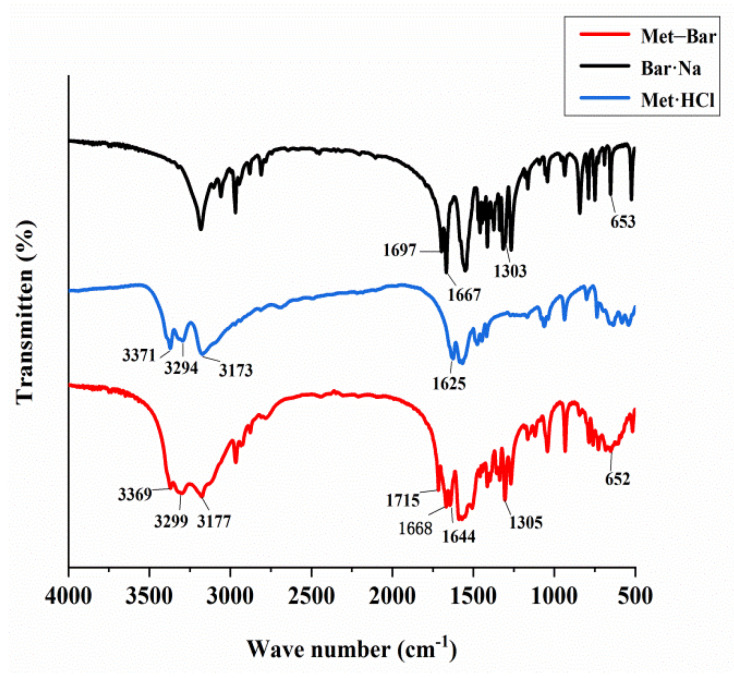
FT-IR spectra of Bar·Na, Met·HCl and Met–Bar.

By means of Fourier-transform infrared spectroscopy analysis, we observed the shifts in the location and intensity of absorption peaks of the characteristic functional groups in the molecules of Met·HCl, Bar·Na, and Met–Bar, testifying whether there were new bonds in the multicomponent crystal of Met–Bar based on their differences, as shown in Figure 10.

The C=O stretching vibration, the carbonyl group of Bar·Na, showed characteristic absorption peaks at 1697 cm^−1^ and 1667 cm^−1^, which corresponded to the absorption peaks of the multicomponent crystal of Met–Bar at 1715 cm^−1^ and 1668 cm^−1^, suggesting the formation of the hydrogen bond between N–H in Met and C=O in Bar. In the fingerprint region of the infrared absorption spectrum, 1303 cm^−1^ and 653 cm^−1^ were the absorption peaks of the C–N stretching vibration in the structure of Bar·Na, and the positions in the multicomponent crystal were 1305 cm^−1^ and 652 cm^−1^, almost unchanged (not involved in hydrogen bonding).

The absorption frequencies of the N–H stretching vibration in Met·HCl were 3371 cm^−1^, 3294 cm^−1^, and 3173 cm^−1^, which were very similar to the vibrational frequencies of the newly formed compound at 3369 cm^−1^, 3299 cm^−1^, and 3177 cm^−1^, indicating that the new compound may also be a salt-type compound with proton transfer like Met·HCl. It agreed with the single crystal X-ray diffraction data. The absorption signal of the C=N stretching vibration was 1625 cm^−1^ in Met·HCl and 1644 cm^−1^ in the structure of the multicomponent crystal, which was shifted to high frequency.

### 2.3. Characterization of the Moisture Sorption Behavior

**Figure 11:**
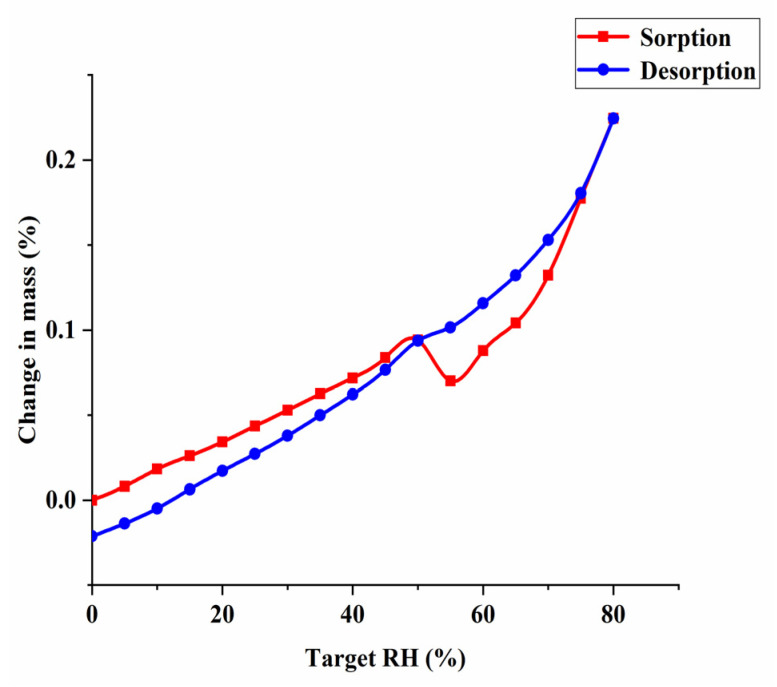
DVS isotherm plot for the multicomponent crystal of Met–Bar.

DVS was used to evaluate hygroscopicity of Met–Bar. Barbiturate drugs with low solubility are mainly made into alkali metal salts to increase solubility, such as barbital sodium, phenobarbital sodium. However, the aqueous solution of sodium salts is usually unstable, easy to hydrolyze when placed at room temperature, so it is often added to powder needles for injection use, limiting the diversity of formulation of this class of drugs [36]. Besides, it is known from the literature that Met is highly hygroscopic. It absorbed a large amount of water after 60% RH, and the weight change was as high as 60% at 80% RH, causing the drug to deliquesce in a short period of time due to the sorption of water [12,13,15]. The structures determined the properties, whether new crystals synthesized by design were able to improve the properties of individual components. Hence, the investigation and verification of the hygroscopicity of Met–Bar was an essential part of our research. As shown in Figure 11, the weight change values of Met–Bar within a certain relative humidity range of 0–80% could be clearly observed to determine the ability of the crystal’s moisture sorption behavior in the air.

However, Met and Bar were combined into a multicomponent crystal, and even when the relative humidity reached 80%, the weight change of the sample was only 0.25%, which well overcame the shortcoming of Met’s high moisture sorption behavior, suggesting that the new compound could be stored stably at ambient RH.

## 3. Conclusions

In this experiment, successful simple synthesis of a multicomponent crystal of Met-Bar demonstrated that the method of designing crystals based on supramolecular synthons with valence bond strength is trustworthy. The crystal structure-based predicted synthon II formed a homodimer and assembled into a larger architecture through the expected one-point synthon III. Additionally, the hydrogen bonds network established by Met and Bar changed the molecular arrangement of individual components and generated a new unit cell packing form, which structurally affected the physicochemical properties of the crystal, reduced the moisture sorption of Met, and increased the stability of Bar.

However, systemic studies of the structure–properties relationship of multicomponent crystals are only rarely conducted at present. Accordingly, the mechanism of multicomponent crystals formation should be discussed in depth to improve crystal screening efficiency. In particular, the main challenge that the emerging research field of multicomponent crystals faces is that the interaction and stacking arrangement between drug molecules can be controlled through other rational and precise crystal designs to achieve targeted changes in the drug melting point, dissolution rate, solubility, bioavailability, biological activity, and other desirable properties to increase the speed of drug development.

## 4. Materials and Methods

### 4.1. Materials

Barbital sodium (Chengdu Chemical Reagent Factory, Chengdu, Sichuan, China) was used as received. Metformin hydrochloride and other solvents and chemicals were purchased from Adamas Reagent Company (Shanghai, China) and used without further purification unless otherwise stated.

### 4.2. Methods

#### 4.2.1. Preparation of Met–Bar

Met·HCl (0.165 g) and Bar·Na (0.206 g) were dissolved in 20 mL boiling methanol and stirred for 1 h. The filtrate was transferred to a 50 mL Petri dish sealed with a parafilm perforated with a few small holes, and finally left undisturbed in a refrigerator at 4 °C to allow cooling crystallization. Crystals were obtained within a week, which were suitable for single crystal X-ray diffraction analysis. For preparing the bulk sample of Met–Bar, 1.650 g Met·HCl and 2.060 g Bar·Na were transferred to 100 mL boiling methanol in a beaker and heated to 90 °C while continuously stirring for 2 h. The filtrate was left to cool at 4 °C with the beaker covered by the parafilm. Large crystals of Met–Bar grew overnight. The crystals were harvested by filtration and dried at 55 °C in a vacuum oven overnight. The crystals were milled for further characterization and formulation.

#### 4.2.2. Single Crystal X-ray Diffraction (SCXRD)

SCXRD data were collected using a Bruker SMART CCD diffractometer (Bruker, Karlsruhe, Germany), with Cu-Kα radiation (λ = 1.54184 Å) at 293 K. Integration and scaling of the intensity data were corrected for empirical absorption effects with the spherical harmonics implemented in the SCALE3 ABSPACK scaling algorithm. The structure was solved with the SHELXS-97 program by direct methods and refined with the least squares minimization using the SHELXL-2014 refinement package. All the non-hydrogen atoms were refined with anisotropic displacement parameters, and the hydrogen atoms were located in the differential Fourier map and refined with isotropic displacement parameters.

#### 4.2.3. Thermal Analysis (TGA and DSC)

The samples (2.307 mg) were heated in a hermetically sealed aluminum pan containing a pinhole on a thermogravimetry analyzer (Q600 SDT, TA Instruments, New Castle, DE, USA) from 25 °C to 500 °C at 10 °C/min under 100 mL/min nitrogen gas.

Differential scanning calorimetry (DSC) was performed with a TA DSC25 (TA Instruments, New Castle, DE, USA). Sample weights of 1.20 mg were heated in Tzero aluminum pans at the scan rate of 10 °C min^−1^ under nitrogen gas.

#### 4.2.4. Powder X-ray Diffraction (PXRD)

PXRD data were collected using a Bruker D8 Advance X-ray diffractometer (Bruker, Karlsruhe, Germany) coupled with Cu-Kα radiation (40 kV, 200 mA, λ = 1.5406 Å). The data for each sample were recorded over the 2θ range from 5° to 50° (step size, 0.02°) at ambient temperature with a scan speed of 5°/min.

#### 4.2.5. Fourier-Transform Infrared Spectroscopy (FT-IR)

Each sample was compressed into a tablet made using the KBr pellet technique and collected by a Nicolet Spectrum FT-IR spectrometer (Nicolet iS10, Waltham, MA, USA) in the 4000–500 cm^−1^ range at the resolution of 4 cm^−1^ with 12 accumulative scans under ambient conditions.

#### 4.2.6. ^1^H NMR Spectroscopy Analysis

^1^H NMR data were collected on a Bruker AVANCE III NMR spectrometer operating at 600 MHz and using DMSO-*d*_6_ as the NMR solvent.

#### 4.2.7. Dynamic Vapor Sorption (DVS)

The materials’ water sorption and desorption profiles were measured using an automated vapor sorption analyzer (Surface Measurement Systems Ltd., Wembley, UK) at 25 °C. The samples of Met–Bar, 26.86 of the mass at the end of the first 0% RH stage, were studied at each step size of 5% RH, following the 0%–80%–0% sorption and desorption cycle. If the weight change within 10 min was smaller than 0.02%, relative humanity (RH) was changed to the next step, with the maximum hold time of 3 h.

## Figures and Tables

**Table 1:** Crystal data and structure refinement for Met–Bar.

Empirical Formula	C_14_H_31_N_7_O_5_
Formula weight	377.46
Temperature (K)	293(2)
Crystal system	Triclinic
Space group	P-1
a (Å)	7.3409(7)
b (Å)	11.1698(10)
c (Å)	13.0980(9)
α (°)	74.680(7)
β (°)	78.624(7)
γ (°)	84.157(8)
Volume (Å^3^)	1014.08(15)
Z	2
ρcalcg (cm^3^)	1.236
μ/mm^−1^	0.791
F(000)	408
Crystal size (mm^3^)	0.12 × 0.1 × 0.09
Radiation	CuKα (λ = 1.54184)
2Θ range for data collection (°)	7.11 to 134.102
Index ranges	−8 ≤ h ≤ 8, −13 ≤ k ≤ 13, −15 ≤ l ≤ 13
Reflections collected	7079
Independent reflections	3627 (Rint = 0.0330, Rsigma = 0.0556)
Data/restraints/parameters	3627/0/278
Goodness-of-fit on F2	1.04
Final R indexes (I ≥ 2σ (I))	R1 = 0.0525, wR2 = 0.1313
Final R indexes (all data)	R1 = 0.0805, wR2 = 0.1552
Largest diff., peak/hole (e/Å^−3^)	0.25/−0.17
CCDC deposit number	2075003

**Table 2:** Hydrogen bonds for Met–Bar.

D–H···A	d(D–H) (Å)	d(H–A) (Å)	d(D–A) (Å)	D–H–A (°)
N(1)–H(1A)···O(2) ^1^	0.84	2.15	2.967	163
N(1)–H(1B)···O(1)	0.83	2.24	2.939	142
N(2)–H(2A)···O(4)	0.88	2.10	2.951	161
N(2)–H(2B)···O(4) ^2^	0.92	1.99	2.908	175
N(4)–H(4D)···O(3) ^2^	0.89	2.19	3.057	166
N(4)–H(4E)···O(5) ^2^	0.89	2.02	2.901	171
N(6)–H(6)···O(1) ^1^	0.80	2.13	2.931	179
O(4)–H(4)···N(7) ^2^	0.86	1.88	2.739	172
O(5)–H(5)···O(3) ^3^	0.78	1.97	2.755	175

Symmetry transformations used to generate equivalent atoms: ^1^ 1 − X, 1 − Y, −Z; ^2^ −X, 1 − Y, 1 − Z; ^3^ −X, −Y, 1 − Z.

## Data Availability

The data presented in this study are available on request from the corresponding author.

## Sample Availability

Samples of the compounds are not available from the authors.
